# Immunologic and nonimmunologic sclerodermal skin conditions - review

**DOI:** 10.3389/fimmu.2023.1180221

**Published:** 2023-07-12

**Authors:** Carmen Bobeica, Elena Niculet, Mihaela Craescu, Elena-Laura Parapiru, Andreea Mioara Corduneanu-Luca, Mihaela Debita, Ana Maria Pelin, Carmen Tiutiuca, Claudiu Ionut Vasile, Alin Codrut Nicolescu, Magdalena Miulescu, Gabriela Balan, Alin Laurentiu Tatu

**Affiliations:** ^1^ Department of Morphological and Functional Sciences, Faculty of Medicine and Pharmacy, “Dunărea de Jos” University, Galaţi, Romania; ^2^ Multidisciplinary Integrated Center of Dermatological Interface Research MIC-DIR (Centrul Integrat Multidisciplinar de Cercetare de Interfata Dermatologica - CIM-CID), “Dunărea de Jos” University, Galaţi, Romania; ^3^ Clinical Medical Department, Faculty of Medicine and Pharmacy, “Dunărea de Jos” University, Galaţi, Romania; ^4^ Department of Plastic Surgery, “Sf. Ioan” Clinical Emergency Hospital for Children, Galaţi, Romania; ^5^ Department of Pharmaceutical Sciences, Faculty of Medicine and Pharmacy, “Dunărea de Jos” University, Galaţi, Romania; ^6^ Clinical Surgical Department, Faculty of Medicine and Pharmacy, “Dunărea de Jos” University, Galaţi, Romania; ^7^ Dermatology Department “Agrippa Ionescu” Emergency Clinical Hospital, Bucharest, Romania; ^8^ Research Center in the Field of Medical and Pharmaceutical Sciences, “Dunărea de Jos” University, Galaţi, Romania; ^9^ Dermatology Department, “Sf. Cuvioasa Parascheva” Clinical Hospital of Infectious Diseases, Galaţi, Romania

**Keywords:** immunological skin conditions, lichen sclerosus (balanitis xerotica obliterans), scleroatrophic lichen, systemic sclerosis, scleromyxedema, scleredema Burschke

## Abstract

Scleroderma-like cutaneous lesions have been found in many pathological conditions and they have the clinical appearance of sclerotic or scleroatrophic lesions. Affected skin biopsies described histopathological changes similar to those of scleroderma located strictly on the skin or those of systemic sclerosis. These skin lesions can be found in inflammatory diseases with autoimmune substrate (generalized morphea, chronic graft versus host disease, eosinophilic fasciitis), tissue storage diseases (scleredema, scleromyxedema, nephrogenyc systemic fibrosis, systemic amyloidosis), metabolic diseases (porphyrya cutanea tarda, phenylketonuria, hypothyroidism, scleredema diabeticorum), progeroid syndromes. Given the multiple etiologies of sclerodermal lesions, a correct differential diagnosis is necessary to establish the appropriate treatment.

## 1 Introduction

The medical literature describes several conditions that mimic the skin lesion of scleroderma located strictly on the skin or of systemic sclerosis (SSc), and can sometimes present clinical elements of both diseases. Some skin lesions are scleroatrophic lesions, others are purely sclerotic. The correct diagnosis of these lesions based on the clinical elements supplemented with paraclinical data is essential in order to correctly direct the therapeutic attitude (1–3). These scleroderma-like disorders can be inflammatory diseases with autoimmune substrate (generalized morphea, chronic graft versus host disease, eosinophilic fasciitis), tissue storage diseases (scleredema, scleromyxedema, nephrogenic systemic fibrosis, systemic amyloidosis), metabolic diseases (porphyria cutanea tarda, phenylketonuria, hypothyroidism, scleredema diabeticorum) (1–3). The presence of sclerodermiform lesions in the graft-versus-host suggests the autoimmune substrate of the lesion (3).

Sclerodermal lesions have also been observed in some inherited diseases, such as progeria and other progeroid syndromes and in fascial dystrophy which is also called stiff skin syndrome (1–3). Given the multiple etiologies of sclerodermal lesions, a correct differential diagnosis is necessary to establish the appropriate treatment (**Table 1**) (3, 4, 104–107).

**Table 1:** Sclerodermiform lesions – clinical and paraclinical characteristics, histological aspects, treatment.

Sclerodermal skin conditions	Clinical and paraclinical characteristics	Histological examination	Treatment
**Systemic sclerosis**	Raynaud’s phenomenon, unilateral/bilateral edema of the hands, skin fibrosis, sclerodermic facies with distinctive sign: “the wrinkled chin sign”, sclerodactyly, visceral fibrosis (1, 4–7)	Perivascular inflammatory infiltrate (lymphocytes, histiocytes), excessive deposition of extracellular matrix (8)	*Vasoactive*: prostanoids (Iloprost, Aprostadil) (9, 10),endothelin receptor inhibitor (Bosentan) (9), Pentoxifylline 30,31 *Corticosteroid* (11, 12) *Immunosuppressants*: cyclophosphamide (11, 13), mycophenolate-mofetil (11) *Antifibrosind agents*: nintedanib (11, 14), imatinib (11), pirfenidone (11) *Monoclonal antibody*: tocilizumab (11) abatacept (8, 15), belimumab (8, 15), rituximab (16–19) *Jakinase inhibitor* (8, 15) *IL13 and IL14 inhibitors* (8, 15) *Hematopoietic stem cell transplantation* (11) *Cannabinoid receptor type 2 agonist:* Lenabasum (11)
**“Sine scleroderma”**	Visceral fibrosis, without skin induration (5, 6)	Complete Type III – similar to SSc (20) Renal biopsy - edema and thickening of the interlobular and arcuate arterioles (21, 22)	Similar to SSc (23)
**Scleromyxedema**	Extensive lichenoid papules, hardened skin spots (face, neck, arms, fingers) (24)	Excessive collagen deposits surrounded by mucin (24)	Intravenous immunoglobulin ± systemic glucocorticoids and thalidomide, bone marrow transplantation and bortezomib or melphalan associated with dexamethasone (25)
**Scleredema adultorum Buschke**	Firm skin (chest, neck), without Raynaud’s phenomenon (24)	Mucin deposits cannot be differentiated from those found in scleromyxedema (24)	Topical or systemic corticosteroids, injection intralesional with corticosteroids/hyaluronidase, penicillin, imunosupresor (methotrexate, cyclosporine), psoralen and phototherapy (UVB, PUVA, UVA1), intravenous immunoglobulin, colchicine, pentoxyfylline, D-penicillamine, interferon-γ, radiotherapy (24)
**Eosinophilic fasciitis** **Schulman’s syndrome**	Bilateral symmetrical edema of the hands, PIIIP and aldolase increased (26)	± Eosinophilia, difficult to differentiate from SSc (26)	First line: *corticosteroids* *Immunosuppressants*: azathioprine, cyclophosphamide, cyclosporine *Dapsone, hydroxychloroquine, Immunoglobulins* (27)
**Sclerodermal lesions associated with neoplasms**	Raynaud’s phenomenon, cutaneous sclerosis, neoplasia, no capillaroscopic changes of the nail bed, negative antinuclear antibodies, positive anti-RNA polymerase III antibodies (28–32)	The same aspect as in scleroderma (33)	Sclerodermiform lesions disappear after neoplasia treatment (34, 35)
**Scleredema diabeticorum**	Diabetic cheiroarthropathy, (associated with type 1 or 2 diabetes) - hardened and thickened skin on the back of the neck, more common in men (36, 37)	Thickened collagen fibers in the dermis, surrounded by mucin accumulation (36, 37)	*Immunosuppressants* (cyclosporine, methotrexate) (36) *Systemic corticosteroid* (36) Doxycycline (36) Colchicine (36) Electron beams radiotherapy (36)
**Acrodermatitis chronica atrophicans**	Unilateral rash on the lower limbs a few years after the tick bite, skin discoloration or red-blue hue followed by diffuse thickening, paresthesias, without pain, anti-Borrelia IgG antibodies in high titer (3, 38–41)	Early active lesion - perivascular inflammatory infiltrate in the dermis with plasma cells, discrete atrophy of the epidermis. Late lesion - epidermal atrophy, interstitial inflammatory infiltrate with plasma cells, rare histiocytes and mast cells (42–44)	Oral Penicillin V for 21 days resulted in the gradual disappearance of the lesion (3, 38–41) 500-1000 mg of Amoxicillin administered orally three times a day; 200 mg of Doxycycline per day in two doses or in a single dose for 14-28 days; 2 grams of Cefotaxime every 8 hours for 14-28 days; 3.000.000 - 4.000.000 UI Penicillin G every 4 hours for 14-28 days (42, 45, 46)
**Linear, circumscribed or generalized morphea**	Pruritic erythema, evolution towards sclerosis with purple halo, joint contracture, increased Il-4 (41, 47)	Perivascular inflammatory infiltrate (Ly TCD4 +) in the dermis, accumulation of collagen up to the reticular dermis (41, 47)	*Topical corticosteroid* (41, 47) Tacrolimus 0,1% - local applications (41, 47) *Immunosuppressants* (methotrexate, mycophenolate-mofetil) (41, 48, 49) UVA1, PUVA, UVB (41, 48, 49)
**Post-drug lipodystrophy**	Scleredema, sclerodactyly, cutaneous sclerosis, Raynaud’s phenomenon, hyperpigmentation, negative antinuclear antibodies, without visceral lesions (29, 50, 51)	Accumulation of eosinophils, macrophages and mast cells (52, 53) Immunohistochemistry has identified a reduction in the volume of adipocytes in the injection site (52, 54)	Lipodystrophy remits or stops after stopping the administration of the causative drug (53) There is no known effective treatment for post-drug dystrophic lesions (54)
**Atrofoderma of Pasini and Pierini**	Hyperpigmentation spots, without skin induration, skin atrophy with depressed relief and sharp edge, on the chest, arms, abdomen,± pruritus, pain, paresthesia. Often associated with localized scleroderma (3, 55)	Collagen fibers sclerosis, hyaline material deposition, fragmented and reduced elastin fibers, attenuated dermis (55)	Ineffective treatment (55) *Modest results:* Tetracycline, doxycycline, penicillin (elevated titers of anti-Borrelia burgdoferi antibodies) (55), topical corticosteroid + hydroxychloroquine (systemic lupus erytematous) (55), calcineurin inhibitors (55) Q-switched Alexandrite laser (hyperpigmented spots) (55)
**Frontal fibrosing alopecia**	Cicatricial alopecia in the strip of the frontotemporal edge of the scalp followed by peripheral fibrosis (56)	Cell apoptosis of the outer epithelial sheath and perifollicular inflammatory infiltrates (Ly) (56)	Ineffective treatment (55) **Poor results:** Dutasteride + local applications of pimecolimus (57), local corticosteroids (57) intralesional triamcinol, 2% or 5% minoxidil local (57), finasteride + 2% minoxidil solution (57), hydroxychloroquine, doxycycline, topical tacrolimus (57), 5-alpha-reducatse inhibitors (57), mycophenolate-mofetil, cyclosporine (57), hair grafts (57)
**Porphyria cutanea tarda**	Vesicles and ulcers on the face, neck and lower limbs with evolution towards sclerosis, hypopigmentation, alopecia, increased level of porphyrin in the urine (3, 29, 50)	Excess collagen in the inflamed dermis, infiltrated with mast cells (29)	Recovery is complete after administration of hepatoprotectors, photoprotectors and immunosuppression with hydroxychloroquine (29, 50)
**Post-toxicity sclerodermal lesions**	Scleroderma, death after ingestion of industrial oil (58)	Lung tissue samples embedded in paraffin showed hypereosinophilia with degranulation (58)	Treatment depends on the multiorgan involvement (59, 60)
**Vulvar lichen sclerosis and balanitis xerotica obliterans**	Inflammation, erythema, cracks, blisters with evolution towards cutaneous sclerosis in the external genital organs (61, 62) Atrophy, hypopigmentation (63–65)	Inflammatory infiltrate (cytotoxic LyT), hyperkeratosis of the basal layer of the epidermis followed by atrophy, destroyed elastic fibers, collagen fibers with modified disposition (61, 62)	*First line of treatment*: for symptomatic sclerotic lesions – topical glucocortizoids, in asymptomatic cases – conservative treatment (65–67) *Second line of treatment*: topical calcineurin inhibitors (tacrolimus and pimecrolimus with topical administration) (68) Immunomodulators (65, 69), topical tacrolimus 0,1% (70, 71) Cystourethroscopy, dilation of urethral strictures (65, 66, 70), ultraviolet phototherapy, intralesionally triamcinolone, ozonated olive oil (65, 69), circumcision in symptomatic phimosis (65, 66) Oral corticosteroid therapy - ineffective (68)
**Nephrogenic systemic fibrosis**	Skin fibrosis in the distal portions of the upper and lower limbs, joint contracture, association with renal failure, negative serological profile for scleroderma (72–78)	Diffuse thickening of the dermis through excessive deposition of type I and III collagen (72–78)	It is recommended to limit the use of the contrast agent, gadolinium, involved in the production of fibrosis (71, 73, 74, 79)
**Progeria infantum (Hutchinson - Gilford syndrome)**	Genetic disease, premature aging, delayed growth (80)	Loss of subcutaneous tissue (80)	Genetic disorder with death around the age of 13.4 years due to heart attack and heart failure - complex management (81).
**Progeria of the adults (Werner’s syndrome)**	Genetic disease, accelerated aging from the age of 30, atrophy and sclerosis of the skin, whitening and hair loss (51, 82, 83)	Loss of subcutaneous adipose tissue (82, 83)	There is no known treatment Is under study: mTOR inhibitors, selective inhibitors of p38 mitogen-activated protein kinase, hiPSCs derived from fibroblasts from Werner syndrome patients, human embryonic stem cell therapy (84–89).
**Scleroderma concomitant with phenylketonuria**	Sclerodermal lesions on the chest and proximal extremities of the lower limbs (37, 51)	It noted greatly increased intracellular level of phenylalanine in the skin and non-specific dermatitis changes (90)	Pegvaliase injectable BH4 synthetic analogue sapropterin dihydrochloride A low-protein diet supplemented with amino acids without l-phenylalanine (91).
**Scleredema with proximal induration of the skin associated with β-hemolytic streptococcal infection**	Distal skin induration on the hands and feet is absent (37)	Mucin deposits cannot be differentiated from those found in scleromyxedema (24)	Antibiotic therapy, intravenous immunoglobulins (92)
**Sclerodermal lesions associated with radiation, vibrations, trauma, silicone implant**	Lesions similar to those of scleroderma (89)	Excessive deposition of collagen in the dermis, in the blood vessel and perivascular walls, numerous lysosomes in the endothelial cells (93)	^*^without data
**Skin sclerosis lesions associated with hypothyroidism**	Severe hyperkeratosis, the hardening and discoloration of the skin on the trunk which then becomes generalized. Is differentiated from other scleroderma lesions and SSc by the distribution, extent and depth of the skin lesion (37, 89, 94)	Accumulation of mucin, hyperplasia of fibroblasts with excessive collagen deposition is observed (94)	The administration of thyroid hormones improved the clinical signs (95)
**Chronic graft-versus host disease**	Sclerodermoid lesions in the form of plaques on the trunk that become generalized and limit the expansion of the chest (37, 51)	Thickening of the reticular dermis and dermal papillae with bundles of collagen fibers increased Presence of melanophages in the dermis (96)	The immunosuppressive treatment with azathioprine in combination with cortisone preparations has proven partially effective (96) 400 mg per day of imatinib mesylate improved the skin lesion (97)
**Sclerodermatous lesions associated with multiple myeloma**	Strengthening of the skin on the entire surface (98)	Skin infiltrated with neoplastic cells (51) and aspects similar to SSc (98)	Amelioration of skin induration after 9 months of treatment with 100 mg thalidomide and 40 mg dexamethasone daily (98)
**Scleroatrophic Huriez syndrome (palmoplantar keratoderma)**	Cutaneous scleroatrophy of the hands and forefoot, sclerodactyly, risk of developing squamous cell carcinoma (51, 99, 100)	Absence of Langerhans cells in the epidermis almost entirely (101) Discrete papillomatosis, acanthosis with aspects of irregularity, parakeratosis, hyperkeratosis, hypergranulosis (102, 103)	Topical retinoids, keratolytics and emollients with 20% urea applied locally (103)

## 2 Discussions

### 2.1 Systemic sclerosis

SSc is a form of scleroderma accompanied by multiple visceralizations. Skin induration is induced by peripheral microvascularculation with autoimmune substrate followed by excessive skin fibrosis. Collagen deposition is also present in the internal organs, especially in the lungs, heart, digestive tract and kidneys (4). The differential diagnosis between SSc and other diseases with scleroderma-like skin lesions is difficult. The authors have shown that the “puckered chin sign” is characteristic of SSc and differentiates it from other sclerodermal lesions (1).

**Figure 1:**
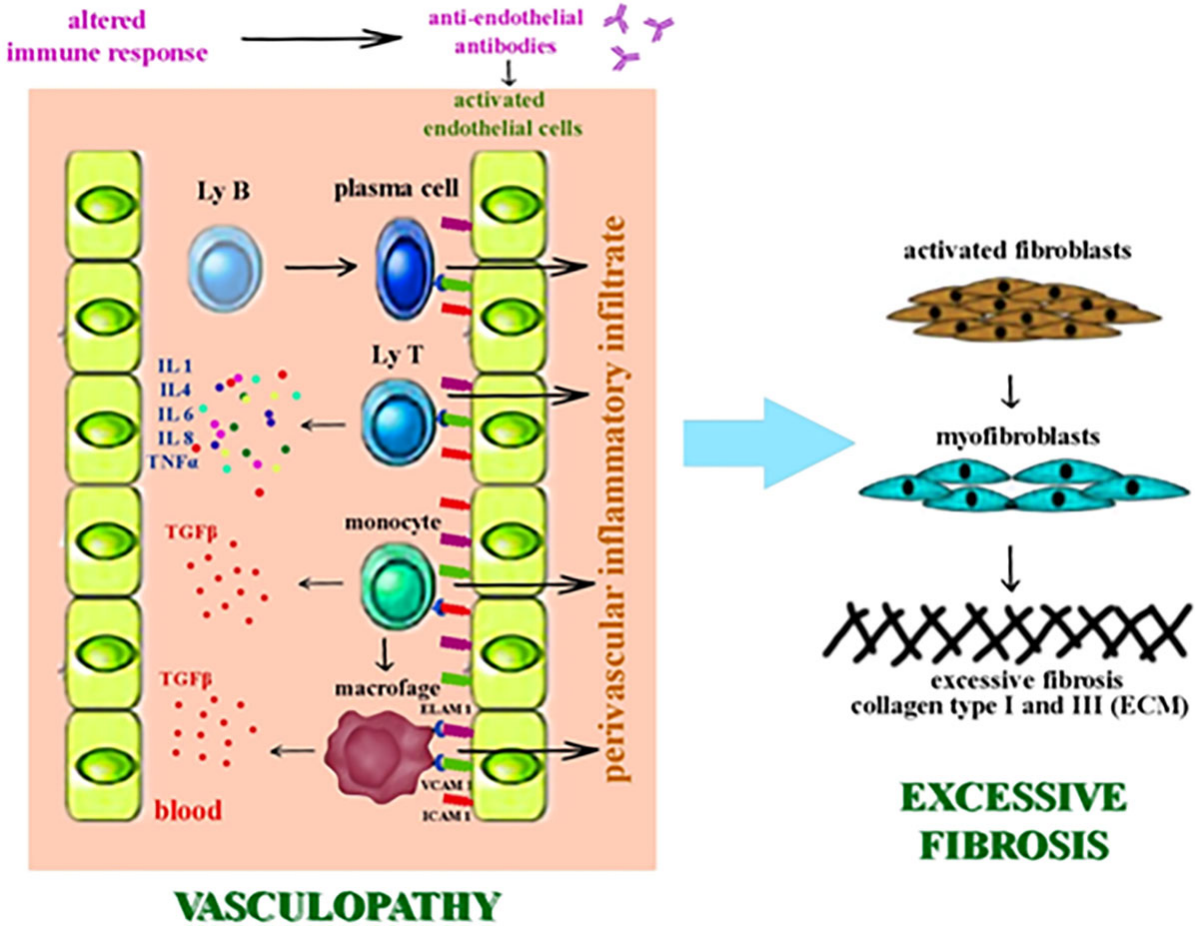
The pathogenic process of SSc - vasculopathy and fibrosis.

The pathogenic process is initiated by the vascular lesion. The endothelial cell is activated by overexpressing the adhesion molecules on its surface: E selectin – endothelial leukocyte adhesion molecule-1 (ELAM-1), vascular cell adhesion molecule-1 (VCAM-1), intercellular adhesion molecule-1 (ICAM-1) (107, 108). Endothelial activation triggers an inflammatory cascade that accumulates proinflammatory cytokines and chemokines, as well as growth factors with fibrogenetic potential (IL1, IL4, IL6, IL8, TNFα, TGF-β). Transforming growth factor β (TGF-β) induces fibroblast hyperactivation which exhibits aberrant profibrotic activity (**Figure 1**) (108–112).

The clinical defining element of SSc is cutaneous induration, except in cases of “sine scleroderma”. Systemic sclerosis without scleroderma (ssSSc) is a very rare form of SSc in which internal organ involvement is not accompanied by skin manifestations or is partially absent, and the immunological profile is abnormal. 3 types of ssSSc are recognized. Type I is also called the complete type because it only shows the damage to the internal organs characteristic of SSc, being completely devoid of skin manifestations, In type II, also called the incomplete type, it shows telangiectasias, calcifications and pitting scars, without sclerodactyly. Type III is called delayed type because the damage to the internal organs characteristic of SSc is followed by complete or incomplete skin lesions. Fibrosis of internal organs without skin changes should suggest ssSSc (20).

**Figure 2:**
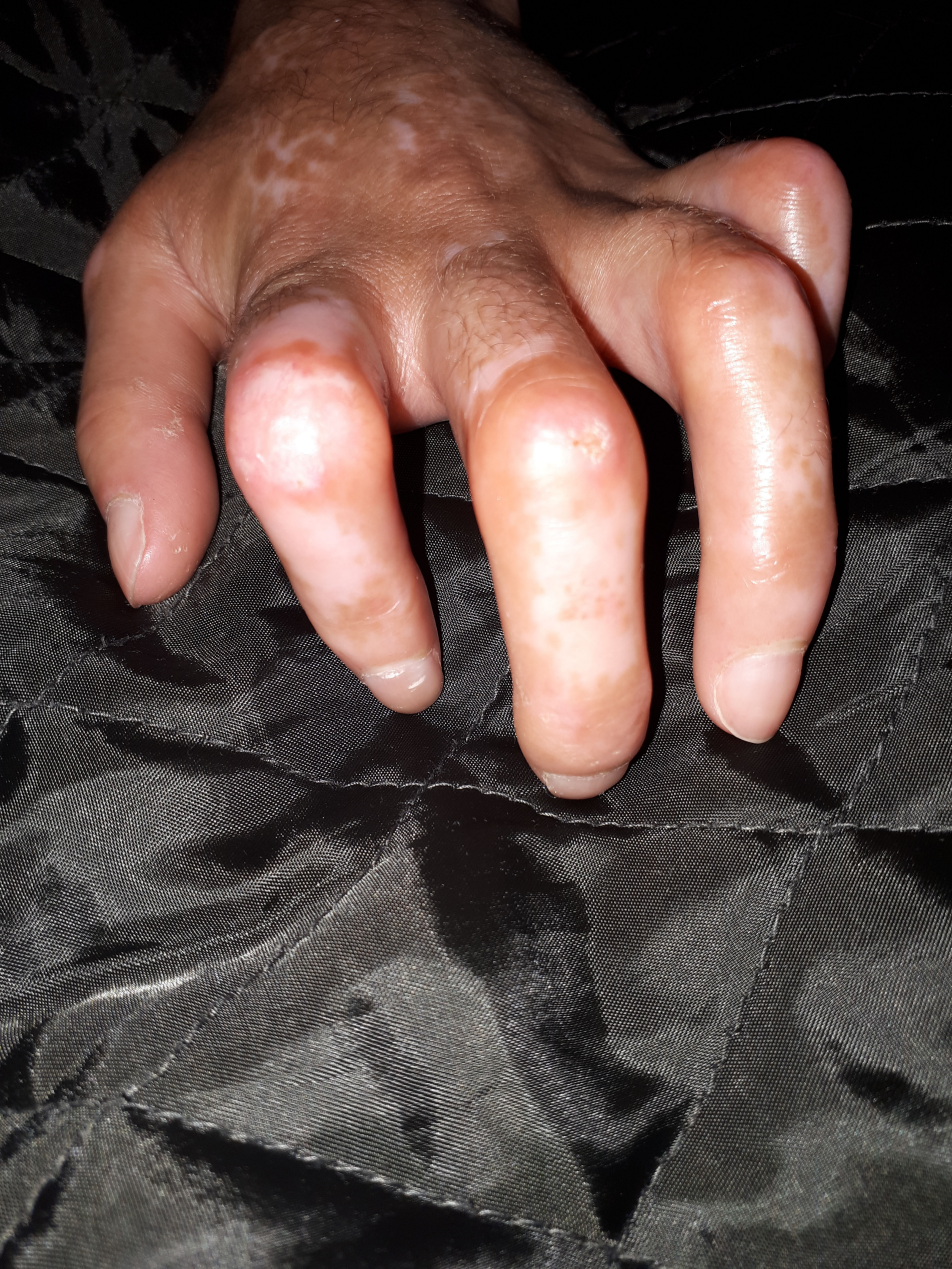
Sclerodactyly.

The skin lesion has three phases; in the first phase, the skin is edematous, waxy, adherent to the subcutaneous planes and loses its elasticity. In the following phases the skin becomes hardened and atrophied. The fingers become stiff, lose their mobility and become fixed in flexion. The hand looks like a claw and sclerodactyly appears (**Figure 2**). Facial skin fibrosis induces the sclerodermal facies characteristic of the disease (5, 6). Raynaud’s phenomenon is present almost constantly in SSc and it is the expression of reversible peripheral vasospasm (7). It is often the first clinical manifestation in SSc and can be found in other autoimmune diseases (9).

The new American College of Rheumatology (ACR)/European League Against Rheumatism (EULAR) classification criteria for the diagnosis of SSc include, besides the clinical criteria (cutaneous sclerosis – major criterion, sclerodactyly, digital ulcers, Raynaud’s phenomenon, telangiectasia), also paraclinical criteria (specific autoantibodies for SSc, a pattern of characteristic capillaroscopic damage, pulmonary damage) (**Table 2**) (113, 114). For the positive diagnosis of SSc, at least 9 points are required to be met (113, 114).

**Table 2:** Diagnostic algorithm in systemic sclerosis (9, 113).

Clinical criteria	Paraclinical criteria
**Cutaneous sclerosis - 9**	**Autoantibodies specific to systemic sclerosis - 3**
**“Puffy fingers”/Sclerodactyly - 3/4**	**Specific damage to capillaries - 2**
**Digital ulcers - 2**	**Pulmonary fibrosis - 2**
**Raynaud’s phenomenon - 3**	**Pulmonary arterial hypertension - 2**
**Telangiectasia - 2**	

Histological examination of the skin after hematoxylin-eosin staining shows the presence of a perivascular inflammatory infiltrate with lymphocytes and histiocytes, vascular lesions and later excessive deposition of extracellular matrix (8). Renal biopsy performed in scleroderma renal crisis in the context of ssSSc, showed edema and thickening intima of the interlobular and arcuate arterioles (21, 22).

Most often the SSc treatment targets the symptoms and includes immunosuppressants, corticosteroids, vasoactive, antifibrosing agents, with unsatisfactory and sometimes modest results (11, 12). Intravenous administration of Iloprost-type prostanoids with vasodilating effect is functional in ameliorating Raynaud’s phenomenon and healing digital ulcers, with possible beneficial effects from topical preparations (used also in diabetic foot ulcers in patients suffering from type 2 diabetes with poor control, based on silver nitrate and Peru balm) (9, 10, 115–117).

Alprostadil is a prostaglandin E1 analogue with a vasodilating and antiplatelet effect used as an alternative to Iloprost in ameliorating Raynaud’s phenomenon and healing digital pulp ulcers (9). The modest response to calcium channel blockers and prostanoids recommends the use of Bosentan, an endothelin receptor inhibitor, which prevents new ulcers and has no effect on healing existing ulcers (9, 10). Pentoxifylline appears to improve digital ulceration due to its vasodilating and anti-TNFα effect, but has not been shown to be effective for Raynaud’s phenomenon (118, 119).

Among immunosuppressants, cyclophosphamide has been shown to be effective in improving respiratory volumes (11, 13). The pathophysiology of SSc has been better understood in recent years. For this reason, new therapeutic targets have been developed. The antifibrosing action of the tyrosine kinase inhibitor, nintedanib, appears to be effective not only on hardened skin but also in secondary pulmonary fibrosis in SSc (11, 14). Two other antifibrosing agents, Imatinib and Pirfenidone, are under study to validate the effect. Its effect is potentiated by the association with mycophenolate-mofetil (MMF) and targets fibroblast growth factor receptors (FGFR3), vascular endothelial growth factor (VEGF) and platelet derived growth factor (PDGF) (11).

Tocilizumab monoclonal antibody is an IL6 signaling inhibitor with reduced effects on skin fibrosis, but with better efficacy for lung damage (11). Although Abatacept treatment has shown encouraging results, further studies are needed for confirmation. Autoimmune response and microvascular impairment are targeted by Jakinase inhibitors, IL13 and IL4 inhibitors, and Belimumab appears to reduce immunoglobulin gene expression and profibrotic pathways (8, 15). Rituximab, an anti-CD20 monoclonal antibody, has shown conflicting results. While some studies have shown that Rituximab improves Rodnan’s skin hardening score with limited effects on forced vital capacity (CVF) (16, 17), other studies have noted unsatisfactory results (18, 19).

New therapies have targeted hematopoietic stem cell transplantation with notable results compared to cyclophosphamide administration. The benefit of autologous transplantation was highlighted only after two years and involves a 10% mortality (11). Favorable results were also obtained after the fat autologous graft (118).

Lenabasum is a new molecule under study and is a cannabinoid receptor type 2 agonist with encouraging results in diffuse SSc (11). A series of hygienic-sanitary measures are meant to alleviate the peripheral vasospasm attacks in the Raynaud’s episodes and to prevent the appearance of digital ulcers. In this regard, it is recommended to protect the extremities from the cold, to stop smoking, to prevent skin lesions, to clean and to dress the skin ulcers (9, 120).

Adjuvant therapies for tissue repair and healing of digital ulcers have been developed, such as: acoustic wave shocks, hyperbaric oxygen therapy, applying of subatmospheric negative that brings the edges of ulcers closer, local applications of vitamin E that accelerates healing or lidocaine to combat pain (118) statins directly or indirectly with their wellknown potential adverse reactions and other potential treatment options with new administration pathways (nanovesicles – exosomes, used as circulating biomarkers) (121–124).

Physical therapy and occupational therapy have improved hand function and especially grip (5, 11). Pet psychotherapy at the time of the Iloprost intravenous infusion has been shown to be effective in reducing anxiety and relieving pain. Fiori observed that the interaction between the dog and the patient with SSc at the time of treatment may also increase adherence to treatment (125). The existence of several types of sclerodermal lesions imposes the need for a differential diagnosis between them and SSc (24). The damage to internal organs in ssSSc equires vasodilatory, immunomodulatory and anti-inflammatory treatment similar to SSc (23).

### 2.2 Scleromyxedema

The skin lesion in scleromyxedema is represented by extensive lichenoid papules that evolve into hardened skin patches on the face, neck and arms with a tendency to extend to the fingers. Histopathological examination shows excessive collagen deposits surrounded by mucin. Usually scleromixedema accompanies other pathologies such as: multiple myeloma, thyroid diseases or monoclonal gamapathies (24). The first line treatment in scleromyxedema remains intravenous immunoglobulin, and the second line includes systemic glucocorticoids and thalidomide alone or in combination with intravenous immunoglobulin. For severe or refractory cases, bone marrow transplantation and bortezomib or melphalan associated with dexamethasone are considered (25).

### 2.3 Scleredema adultorum Buschke

Similar to scleromixedema, the skin induration in scleredema adultorum Buschke is accompanied by mucin deposits, but the topography is slightly different. The hard skin is limited to the upper chest and neck and lacks extension to the fingers. Scleredema Burschke is associated with diabetes and respiratory infections (24). Thoracic damage can interfere with pathologies and pre-existing conditions, in terms of influencing, status changing and worsening (126, 127).

The response to treatment is often absent, other times the lesion resolves partially spontaneously (24). It can be differentiated from SSc by the clinical appearance of the skin lesion and by the almost constant presence of the Raynaud’s phenomenon in SSc. The immunological abnormalities and the capillaroscopy appearance characteristic of SSc direct the diagnosis. Histological examination is not very useful for diagnosis (24). Treatment includes topical or systemic corticosteroids, intralesional injection with corticosteroids or hyaluronidase, immunosuppressive treatment with methotrexate or cyclosporine, psoralen and phototherapy (UVB, PUVA, UVA1), intravenous immunoglobulin, antibiotics (penicillin). Other therapeutic options include: colchicine, pentoxyfylline, D-penicillamine, interferon-γ, and radiotherapy brings a benefit to the hardened skin through the apoptosis of aberrant fibroblasts in the dermis under the action of ionizing radiation (128).

### 2.4 Eosinophilic fasciitis Schulman

Eosinophilic fasciitis (Schulman’s syndrome) is difficult to differentiate from SSc because histology can lead to confusion (26). SSc fibrosis extends from the dermis to the hypodermis and can sometimes affect the fascia of the muscle and the muscle to the bone. On the other hand, in eosinophilic fasciitis the fibrosis of the muscular fascia can extend to the dermis. Differential diagnosis is even more difficult when eosinophilia is absent, a fact quite common in eosinophilic fasciitis or transient when present. In eosinophilic fasciitis the edema of the hands is bilaterally symmetrical, while in SSc the edema may be bilateral or unilateral. Elevated blood levels of type III precollagen peptides (PIIIP) and aldolase appear to be characteristic of eosinophilic fasciitis (26).

Corticosteroids represent the first line of treatment (7). Good results were also obtained after the administration of immunosuppressants (azathioprine, cyclophosphamide, cyclosporine), dapsone, hydroxychloroquine and immunoglobulins (27).

### 2.5 Sclerodermal lesions associated with neoplasms

The sclerodermiform condition has often been associated with neoplasms. Rodisco and colleagues identified the presence of Raynaud’s phenomenon and cutaneous sclerosis associated with colorectal carcinoma in a 60-year-old patient. They note that cutaneous sclerosis could be a paraneoplastic manifestation (28, 29). This aspect was also taken into account in the case of the Mekel diverticulum with neoplastic risk (30). Similarly, Monfort and colleagues identified several cases of SSc associated with neoplastic disease. In 2010, a study by Monfort noted two cases of SSc associated synchronously with ovarian cancer with peritoneal metastases and one case of SSc that associated colon cancer with ovarian metastases one year after onset. They classified these cases of SSc as paraneoplastic syndrome and did not notice increases in antinuclear antibody titers or capillaroscopic alterations of the nail bed (31, 32). Undescended ovaries with neoplastic potential could be associated with SSc (129). A few years later (2018), Monfort and his study team observed that SSc has an increased risk of neoplasia, especially when associated with an increased titer of anti-RNA polymerase III antibodies (31, 32). The diagnosis of sclerodermiform syndrome requires additional investigations to identify a possible background neoplasia. Studies have shown that sclerodermiform lesions disappear after neoplasia treatment (34, 35).

The literature records cases of neoplasia associated with sclerodermiform lesions, such as myeloma accompanied by paraneoplastic syndrome represented by skin lesions with a partial lichenoid appearance (130), or skin sclerosis induced by neoplasm of the breast, cervix, ovary, stomach, esophagus, melanoma and nasopharynx. Scleroderma can appear against the background of neoplasia through the hormones and cytokines released by the tumor tissue that induce cytotoxic effects and the formation of autoantibodies. Sometimes the tumor appears against the background of systemic sclerosis or in the context of immunosuppressive treatment. The histological examination of sclerodermatous lesions is the same as that of SSc (33).

### 2.6 Scleredema diabeticorum

**Figure 3:**
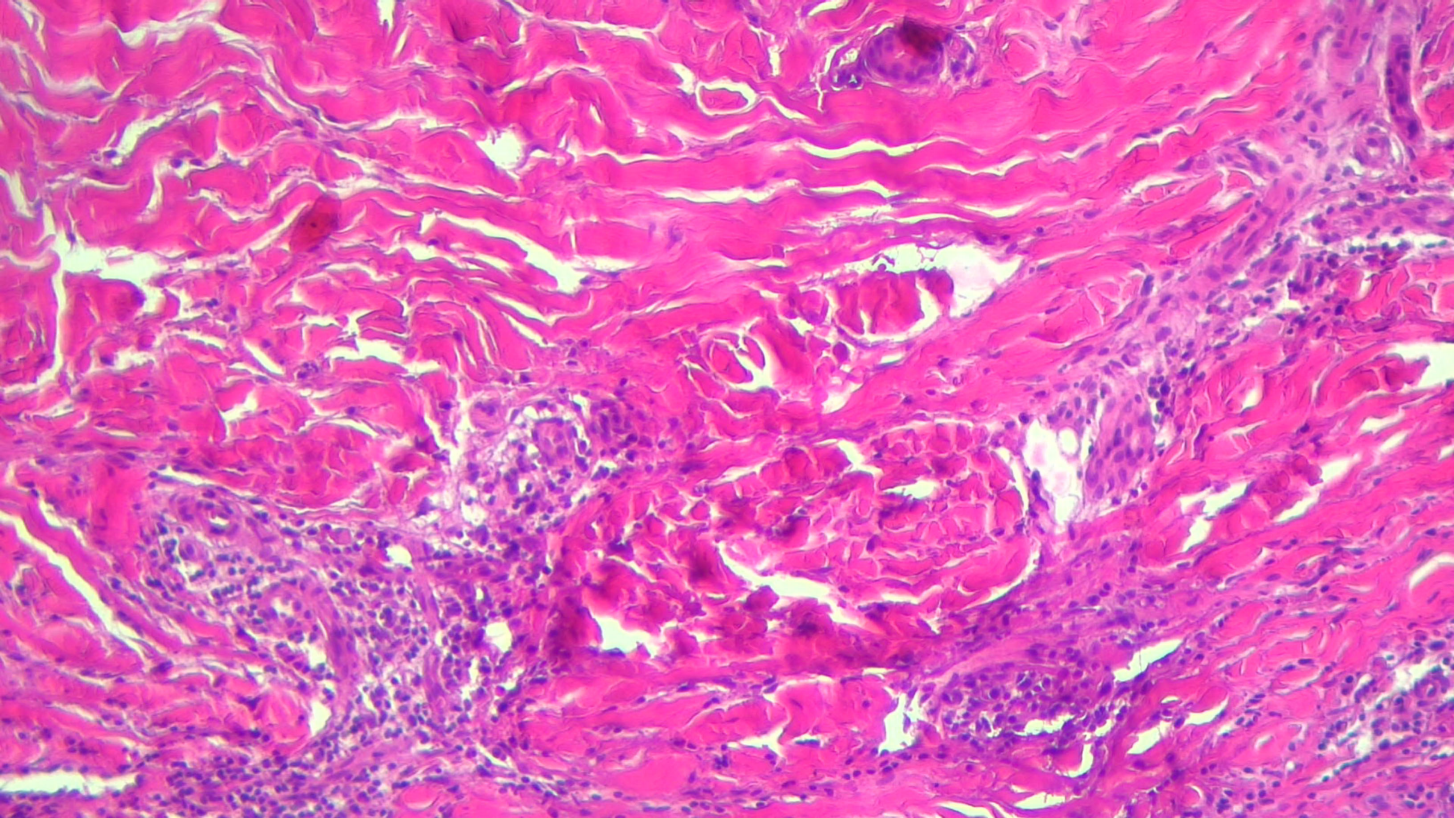
Scleroderma-like changes with thickened deep dermis collagen bundles and chronic inflammation.

Scleredema diabeticorum is considered to be a skin syndrome associated with type 1 or type 2 diabetes in the context diabetic cheiroarthropathy (36, 37). It is rarely seen and is manifested by the strengthening and stiffening of the skin and subcutaneous tissue on the nape of the neck and in the upper region of the posterior thorax, without manifestations in the spectrum of collagenosis. It occurs more frequently in men and is favored by poor glycemic control and obesity. Patients complain of pain and tight skin sensation that limits the range of motion. Skin biopsy revealed thickened collagen fibers in the dermis surrounded by mucin clumps that thicken the dermis as a whole (**Figure 3**). The administration of immunosuppressants (cyclosporine and methotrexate) has not been shown to be effective. Systemic corticosteroid therapy is partially effective, but its duration of administration has been limited due to the risk of glycemic imbalance. Also, a partially favorable response was observed after electron beams radiotherapy and the administration of doxycycline and colchicines (36, 131).

### 2.7 Acrodermatitis chronica atrophicans

Acrodermatitis chronica atrophicans is a rare scleroatrophic lesion present in the tertiary stage of Lyme disease. Diagnosis is difficult because the skin lesion appears several years after the tick bite. Unilateral and often lower limb localization after a rash-like is a valuable clue to the diagnosis. Diffuse thickening characteristic of chronic acrodermatitis atrophicans was preceded by skin discoloration and red-blue tint. The lesion may be accompanied by paresthesias, and pain may be absent. Elevated serum titers of IgG-type anti-Borrelia antibodies are suggestive. Oral doses of Penicillin V administered for 21 days led to the gradual disappearance of the lesion (3, 38–41). The etiological agent is represented by two genus species of Borrelia burgdoferi, Borrelia afzelii and Borrelia garinii. This form of Lyme disease is more common in Europe and can rarely be identified in the United States. It has been observed that acrodermatitis chronica atrophicans may be accompanied by lymphocytomas (3, 40). The early active lesion shows perivascular inflammatory infiltrate in the dermis with plasma cells, discrete atrophy of the epidermis. The late lesion shows epidermal atrophy, interstitial inflammatory infiltrate with plasma cells, rare histiocytes and mast cells (42–44). Other treatment regimens include: 500-1000 mg of Amoxicillin administered orally three times a day or 200 mg of Doxycycline per day in two doses or in a single dose for 14-28 days. If intravenous injection administration is necessary, there are two options, 2 grams of Cefotaxime every 8 hours for 14-28 days or 3.000.000 – 4.000.000 UI Penicillin G every 4 hours for 14-28 days (42, 45, 46).

### 2.8 Morphea

**Figure 4:**
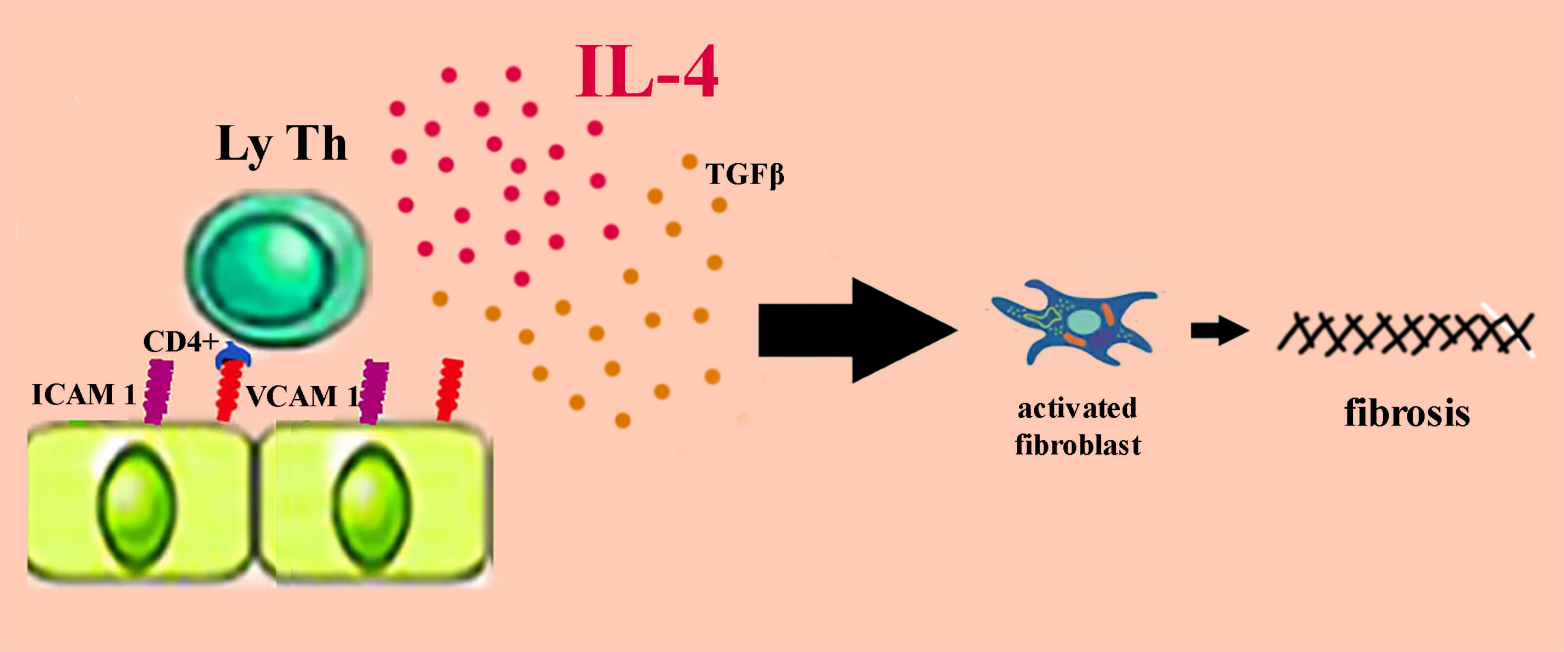
The pathogenesis of morphea.

Scleroatrophic manifestations are also present in morphea, a localized form of scleroderma. The initial skin lesions are erythematous, slightly itchy and progress to sclerotic lesions surrounded by a purplish halo. Early lesion biopsy shows a perivascular accumulation of inflammatory cells in the dermis, predominantly LyTCD4 + and plasma cells (**Figure 4**). The accumulation of LyT is favored by the overexpression of ICAM-1 and VCAM-1. In morphea, the level of IL4 provided by LyTCD4 + is often elevated, and high titers of antinuclear antibodies present in generalized morphea indicate the autoimmune substrate of the disease. Similar to SSc, high levels of IL4-induced TGF-β activate the fibroblast which is responsible for excessive collagen production (41, 47). The old lesions in the morphea acquire a sclerotic appearance by the accumulation of collagen in the form of bundles up to the reticular dermis where the blood vessels and eccrine glands are incorporated. The linear, circumscribed and generalized shapes of morphs can extend to the deeper layers producing joint contracture. When scleroatrophic lesions are severe, magnetic resonance imaging is useful for assessing their depth and for identifying tenosynovitis, myositis, or thickening of the fascia. Morphea management depends on the subtype of the disease, the degree of activity, and the depth of the scleroatrophic lesions. Early intervention in severe forms of morphea is essential to limit deformities, joint contractures and sclera-skin atrophy. The first-line treatment for superficial forms of morphea remains topical corticosteroid administration for 3 to 4 weeks. As an alternative to treatment, tacrolimus 0.1% in local applications is effective in treating superficial lesions in circumscribed morphea (41, 47). In generalized morphea with deep skin lesions, treatment is supplemented with methotrexate or mycophenolate mofetil in combination with A1 ultraviolet (UVA1) phototherapy. This type of ultraviolet has a lower risk of sunburn and has a better penetration than B ultraviolet. Phototherapy with UVA1 can be replaced with broadband ultraviolet A (UVA), psoralen with long wave ultraviolet (PUVA) or ultraviolet B (UVB) in narrow band (41, 48, 49). If the morphea is deeply extended to the muscular and skeletal planes, phototherapy is ineffective (41). Recent papers showed the induction of morphea by SARS-CoV-2 infection, following the COVID-mRNA vaccine or associated with other autoimmune diseases (132–137).

### 2.9 Post-drug lipodystrophy

Numerous authors have reported sclerodermal lesions over time after chemotherapy, such as docetaxel and bleomycin. Bleomycin produces sclerodactyly with scleredema, cutaneous sclerosis, Raynaud’s phenomenon and hyperpigmentation. Also, Docetaxel induces scleroderma-like lesions, edema of the subcutaneous tissue in the limbs, erythematous spots with varying intensity from pale red to purplish, and even bullous lesions (29, 50).

Sharawar and colleagues presented the case of two sibling children aged 4 and 2 who received an unknown dose of intramuscular injectable triamcinolone administered by an unqualified person for 4 consecutive days. In the administration area, on the buttocks and arms bilaterally, there were depressed and slightly hypopigmented focal areas of the skin. No known causes of these skin lesions have been identified in the history and have been interpreted as localized lipodystrophy induced by local cortisone injection. Similarly, the Japanese study conducted by Hisamichi reported that lipodystrophy occurred a short time after intramuscular injection of corticosteroids. Immunohistochemistry has identified a reduction in the volume of adipocytes in the injection site. Activation of macrophages at the injection site is thought to increase cytokine levels, which amplify local lipid catabolism and block local lipid synthesis. Lipid atrophy at the injection site is also favored by decreased local blood flow (52, 54).

Subcutaneous lipodystrophy has also been reported after intramuscular injection of gentamicin. A 9-month-old boy showed a depressed area on his left buttock that corresponds to the injection site of a single dose of gentamicin. The area of lipodystrophy was progressively established within 15 days of the injection and was reported by the child’s parents 1 month after treatment. The overlying skin remained unchanged. In both cases, the parents of the children with lipodystrophy refused the biopsy of the lesion. Other drugs, such as insulin, penicillin, injectable steroids, have been implicated in the development of localized lipodystrophy (54, 138) or bullous reactions followed by local dystrophy and difficult local treatments (139, 140). Rarely, scleroderma-like skin lesions have been observed after administration of paclitaxel, gemcitabine, peplomycin, cocaine, uracil-tegafur, methysergide, interferon-β1a, vitamin B12, vitamin K. For the differential diagnosis between these sclerodermiform lesions and SSc, the absence of specific autoantibodies and visceral damage is taken into account, but also the characteristics of the skin lesion (51). Lipodystrophy was also observed at the insulin injection site under two aspects, lipoatrophy and lipohypertrophy. Lypoatrophy presents itself as a depressed, scar area as a result of the destruction of adipose tissue. The biopsy of the lesion identifies the presence of eosinophils, macrophages and mast cells (52, 53). Lypohypertrophy looks like a prominent area, hardened, with a “rubbery” appearance, and sometimes with a low, softer consistency Lipodystrophy remits or stops after stopping the administration of the causative drug (53). There is no known effective treatment for post-drug dystrophic lesions (54).

### 2.10 Atrofoderma of Pasini and Pierini

Atrophoderma is a rare skin lesion that results in dermal atrophy. It was first described in 1923 by Pasini, who called it “progressive idiopathic atrophoderma.” A few years later, in 1936, Pierini and Vivoli noticed an association with morphea in plates. The differential diagnosis between atrophoderma and scleroderma is extremely difficult or even impossible (55). The etiology of atrophoderma is unknown, although more than half of the patients included in the study by Buechner and Rufli showed high titers of anti-Borrelia burgdoferi IgG antibodies without the presence of IgM antibodies (55, 141).

Clinically, the atrophoderma of Pasini and Pierini comes in the form of multiple hyperpigmented spots, rarely hypopigmented, without skin induration and without obvious inflammation. Sometimes, atrophy may be present as a single lesion. The lesions have different sizes, with slightly depressed relief and a sharp edge. They are present on the chest, first on the back and then on the front, after which they extend on the arms and abdomen. Sometimes the lesions are itchy, painful and can be accompanied by paresthesia (55).

As early as 1998, Jablonska associated the clinical appearance of progressive facial hemiatrophy with Pasini-Pierini atrophoderma and noted a resemblance to localized scleroderma. Jablonska noted that these two types of injuries are often concomitant (3). In atrophy, the histological examination shows a slight atrophy of the dermis, usually without epidermal changes. Derm thickness is reduced by fragmentation, atrophy and sclerosis of the collagen fibers, followed by impregnation with hyaline tissue. The histological aspect is completed by the fragmentation and reduction in size of the elastin fibers. Ectasia of blood vessels in the superficial layers and perivascular accumulation of an inflammatory infiltrate without affecting the sebaceous and sweat glands have been identified less frequently. No treatment has been shown to be effective in atrophy. The results were inconsistent after therapy with tetracycline, doxycycline or penicillin in patients with elevated titers of anti-Borrelia burgdoferi antibodies. Patients who also associated with systemic lupus erythematosus responded fairly well to topical corticosteroid therapy combined with hydroxychloroquine. After topical administration of calcineurin inhibitors, only occasional improvement was observed. Q-switched Alexandrite laser appears to be effective for hyperpigmented spots (55).

### 2.11 Frontal fibrosing alopecia

Frontal fibrosing alopecia is another scleroatrophic skin lesion that affects the skin of the forehead near the fronto-temporoparietal line of the hair. It was originally called postmenopausal frontal fibrosis alopecia because it was seen in postmenopausal women. The authors’ opinions differ on the framing of frontal fibrosing alopecia. In 1994, Kossard first described it and considered that it as a particular form of lichen planopilaris. Several authors classified frontal fibrosing alopecia to be a distinct form of the disease. The incidence of this form of alopecia has increased in recent years probably due to exposure to a trigger that remains unknown (56).

The onset of menopause indicates the involvement of a hormonal factor in the etiology of the disease. Moreover, antiandrogen therapy has been shown to be effective, but hormone replacement does not affect the disease. Frontal fibrosing alopecia occurs not only in menopausal women, but also in men, without a hormonal imbalance (57). This scarring alopecia appears to be an autoimmune disease that results in the destruction of the hair follicle in the infundibular and isthmic area and the melanocyte in the upper follicle. TGFβ could induce an epithelial-mesenchymal transition through which the follicle transforms into fibroblasts. Similar to the pathogenic chain of systemic scleroderma, fibroblasts and epithelial cells are differentiated into myofibroblasts that promote the growth of the extracellular matrix (56).

Histological examination of frontal fibrosing alopecia reveals cellular apoptosis of the outer epithelial sheath, more important than that of lichen planopilaris, and a perifollicular inflammatory infiltrate with lymphocytes, lower than that of lichen planopilaris. The hair is affected in all phases of the cycle. The lesion progresses to cicatricial alopecia with a band-like appearance at the frontotemporal edge of the scalp. In this area the hair follicle is destroyed by the appearance of an area of peripheral fibrosis. No truly effective treatment was found. Local administration of corticosteroids, intralesional injection with triamcinol, local applications with 2% or 5% minoxidil, hydroxychloroquine, doxycycline and topical tacrolimus with poor results were attempted. Administration of 5-alpha-reductase inhibitors appears to be effective. The combination of 0.5 mg/day dutasteride for 6 months and local applications of pimecolimus for 3 months was able to stabilize the disease. Also, 2.5 mg/day finasteride combined with 2% minoxidil solution over a period of 18 months had a favorable effect. There is insufficient evidence for mycophenolate mofetil or cyclosporine, and the side effects are discouraging. With cosmetic effect, hair grafts are recommended after the disease has stabilized. It should be remembered that spontaneous stabilization of frontal fibrosing alopecia is possible (57).

### 2.12 Porphyria cutanea tarda

Porphyria cutanea tarda is another clinical entity with sclerotic potential on the skin. Calado and colleagues reported the case of an elderly black patient with sclerodermal lesions on the background of late cutaneous porphyria secondary to viral hepatitis C. The level of porphyrin in the urine was very high. Exulcerative and vesicular skin lesions on the face, neck, and lower limbs progressed to sclerosis, hypopigmentation and alopecia and completely recovered after administration of hepatoprotectants, photoprotectors, and hydroxychloroquine immunosuppression (29, 50). Scleroderm lesions have been identified in other cases of late cutaneous porphyria without the presence of hepatic viral infection, sometimes in the presence of hemochromatosis and appear to be associated with female sex (3, 29). Histopathological examination showed excessive collagen deposition in the hardened and infiltrated inflammatory dermis with a predominance of mast cells (29).

### 2.13 Post-toxicity sclerodermal lesions

Skin changes in scleroderma were identified in 1981, after the consumption of denaturing rapeseed oil in the Spanish population of Madrid. 20.000 patients had multi-organ damage called “Spanish oilseed rape disease” or “toxic oil syndrome”. Rapeseed oil for industrial use has been refined to remove aniline contained in a percentage of 2%. It was later mixed with edible oil and marketed as a food product. More than 300 consumers died and 13% of survivors had scleroderma (58). Lung tissue samples embedded in paraffin showed hypereosinophilia with degranulation (58). The treatment in toxic oil syndrome depends on the multiorgan involvement (59, 60).

### 2.14 Vulvar lichen sclerosis and balanitis xerotica obliterans

Data from the literature record several names for the scleroatrophic lichen lesion: lichen sclerosus, leukoplakia or kraurosis vulvae (63). Vulvar lichen sclerosis describes the same lesion as balanitis xerotica obliterans (BXO), but is present in women (70, 142). In 1881, Hallopeau described such a lesion which, later in 1976, was called lichen scleros (by The International Society for the Study of Vulvovaginal Disease) (63).

BXO, also called penile lichen sclerosus, is a chronic inflammatory disease with autoimmune substrate that progresses to cutaneous sclerosis of the external genitalia. It is a sclerodermal condition and is synonymous with scleroatrophic lichen in men. The skin lesion involves the foreskin, penis and the external urinary meatus. In 1928, Stuhmer noted that BXO is similar to the scleros lichen that affects the vulva in women (70, 142). Histopathological examination of BXO and vulvar lichen sclerosis is identical, suggesting that they are the same pathology (142).

Statistics show that the incidence of BXO has increased in boys in recent years and often leads to secondary phimosis. Persistent physiological phimosis despite conservative treatment may direct the diagnosis to secondary BXO phimosis. The prevalence was estimated to be between 0.1% and 0.4% among boys in Germany and 0.07% in boys under the age of 10 in the United States (70). Balanitis is often asymptomatic in the early stages. During evolution, the penis and foreskin acquire an erythematous appearance or hypopigmented white areas may appear (66).

The etiology of BXO is unknown, but it has autoimmune determinism and is also associated with other autoimmune diseases: vitiligo, alopecia areata, pernicious anemia, autoimmune thyroiditis (61, 63, 70, 143–146). Although the etiology of the disease is not known, the literature records the predisposing role of the genetic field on which some local infections or traumas can act. There is no clear evidence, but it is assumed that some infections with bacteria, viruses and spirochetes may be determining factors, especially when there is a predisposing genetic background. BXO can progress to squamous neoplasm (70). Local irritation and chronic inflammation promote the progression of dermatoses (66, 68).

Clinical examination of BXO in the early stages shows inflammation and erythema of the preputial frenulum, cracks and blisters from which serous fluid leaks. Late, the scarring of the lesion leaves a whitish circular area of the foreskin, and the glans has a thickened epithelialization on its surface and may be accompanied by meatitis. Histopathological examination confirms the diagnosis and reveals abundant inflammatory infiltrate with cytotoxic LyT and autoantibodies in the extracellular matrix of the skin lesion, hyperkeratosis of the basal layer of the epidermis followed by atrophy, destruction of elastic fibers and change in collagen fiber disposition (61, 62). Some authors use the term lichen sclerosus for both the sclerotic lesion of women and men. Studies have reported a variable prevalence of the disease, between 0.1% and 3% (55, 64). While some studies show that the female-male ratio of this lesion varies from 1:1 to 10:1 (63, 64), other authors estimate an almost identical prevalence between the sexes (70).

The pruritic mucocutaneous lesion of the scleros lichen is often genital and has the appearance of atrophy and hypopigmentation (63–65). The onset is between 8-13 years and 50-60 years. Genital localization was recorded in 85-98% of cases. Cases of scleros lichen with localization to the oral mucosa have been reported very rarely (63, 64). In evolution, the morpho-functionality of the skin and mucous membranes is affected. Sclerotic lesions leave vaginal scars in women, and in men they can cause phimosis (63, 65). Difficult retreat of the foreskin from phimosis leads to urinary disorders and long-term sexual disorders. Neglecting them can be complicated by dysuria, urethral meatus strictures, urinary retention and even kidney failure (65, 66). The risk of complication with a neoplasm requires monitoring of lesions (63). It is estimated that 3-6% of women and 2-8% of men progress to squamous cell carcinoma. The diagnosis takes into account the clinical aspect and the histopathological confirmation of the lesion (65, 66, 84). Human papillomavirus (HPV) infection is involved in 50% of squamous cell carcinoma located in the penis (70, 147).

Neill and colleagues (2002) described scleros lichen in girls as an 8-shaped perineal lesion resulting from the confluence of lichenoid areas on the vulva or anus. Pain, itching, and difficult defecation are often reported when anal fissures occur (69). Lichen sclerosis requires differential diagnosis with Zoon balanitis, also called plasma cell balanitis which has the appearance of a flat red plaque sometimes accompanied by smaller spots. Each time histopathological examination is required for a definite diagnosis (66, 67). The white plaque in leukoplakia is very similar to lichen sclerosis. Irritation between the foreskin and the glans can induce a neoplastic transformation that requires biopsy again (66). Contact dermatitis and penile psoriasis can cause lichen sclerosis-like lesions (66, 67). Moreover, lichen sclerosus/scleroatrophic can be associated with various comorbidities, a situation also found in other pathologies with an immunological component, such as psoriasis (148, 149).

In asymptomatic BXO the treatment is conservative. For symptomatic sclerotic lesions, topical glucocortizoids are the first therapeutic option (65–67) and are effective in over 90% of cases (70, 150). Oral corticosteroid therapy is not effective (68). Topical calcineurin inhibitors, such as tacrolimus and pimecrolimus with topical administration, are indicated as line 2 treatment (68).

Ultraviolet phototherapy, intralesionally administered triamcinolone, immunomodulators, ozonated olive oil are other treatment options (65, 69). Circumcision is necessary in phimosis accompanied by symptoms and must be preceded by topical corticosteroid therapy with anti-inflammatory visa (65, 66).

Local administration of tacrolimus (ointment with concentration of 0.1%) with immunosuppressive effect after circumcision has been shown to be effective in 91% of complicated BXO cases affecting the urinary meatus and glans (70, 71). It is essential to practice circumcision no later than 1 year after diagnosis to prevent urethral strictures (65, 70). Determining the location and severity of urethral lesions requires cystourethroscopy, and the presence of urethral strictures requires dilation, internal urethrotomy with direct visualization, meatotomy, and in case of failure, urethroplasty, meatoplasty or preputioplasty are performed. Interventions are recommended during the remission period of the disease (65, 66, 70). Early diagnosis and management of BXO is followed by disease regression in 92% of cases (70).

In 2018, Naciri and Benzekri reported the case of an 8-year-old girl with two autoimmune skin lesions, extragenital scleroatrophic lichen and vitiligo. This association is rare and causes the destruction of melanocytes. The scleroatrophic lichen was present in the form of atrophic papules of white-pearl color arranged in plates and were located on the abdomen, interscapular and at the knees. Topical corticosteroid therapy was very effective both for the infiltrative lesion, which proved to be scleroatrophic lichen at the skin biopsy, and for vitiligo (79, 151). Topical administration of cortisone is equally effective for genital scleroatrophic lichen (152, 153).

### 2.15 Nephrogenic systemic fibrosis

In 2021, Sarwal and Gnanasekaran presented the case of a female patient with a chronic end-stage kidney disease in a terminal stage and secondary anemia associated with multiple comorbidities: diabetes mellitus with neuropathy, osteodystrophy in the context of secondary renal hyperparathyroidism, peripheral venous insufficiency, chronic viral hepatitis C. The female patient has been proposed for kidney and liver transplantation, being under hemodialysis since 2001. Diffuse thickening of the dermis at the extremities of the upper and lower limbs was observed during magnetic resonance imaging. Since 2008, the female patient has been complaining of bone pain and skin hardening of the forefoot. In this context, the serological profile for scleroderma with a negative result was determined. Subsequently, skin biopsy showed nephrogenic systemic fibrosis (72).

Several authors have observed thickening and hardening of the skin from the extremities of the upper and lower limbs accompanied by excessive fibrosis in patients with acute and chronic renal failure. It has been called nephrogenic systemic fibrosis and is characterized by rapid progression to joints contractures that immobilize the patient in a wheelchair. Cases have been reported in which cutaneous fibrosis spread to the subcutaneous layer and striated muscles, lungs, myocardium and pericardium. The etiology remains unknown, although some authors have implicated hemodialysis and aromatic amines entering the bloodstream as a trigger factor III (73–78). However, nephrogenic systemic fibrosis has also been identified in patients with chronic renal failure who were not on dialysis. A number of unknown determinants are thought to activate an aberrant fibrocyte responsible for TGF-β overexpression and excessive collagen deposition type I and III (73–78). Other authors believe that gadolinium used as a contrast agent may be involved in the production of fibrosis, which is why it is recommended to limit its use (71, 73, 74, 79).

### 2.16 Other sclerodermiform conditions

Skin areas that mimic scleroderma are also found on the chest of patients with progeria infantum also known as Hutchinson - Gilford syndrome. This very rare disease is characterized by premature aging and delayed growth of the individual, senescent appearance of the entire skin and loss of subcutaneous tissue (80). It is a genetic condition caused by mutation of the lamin A gene that causes premature aging and death around age 13.4 from heart attack and congestive heart failure (81).

Another progeroid syndrome is Werner’s syndrome, called “Progeria of the adults” by Thannhauser in 1945 because it begins in adulthood. Werner’s syndrome is a rare autosomal recessive genetic disease that results in premature aging due to connective tissue damage (51). Aging is accelerated from the age of 30, the skin atrophies, sclerosed skin appears, subcutaneous adipose tissue is lost, hair turns white and falls out (82, 83). There is no known treatment for Werner syndrome, but several therapeutic options are being studied, mTOR inhibitors, selective inhibitors of p38 mitogen-activated protein kinase (MAPK), human induced pluripotent cells (hiPSCs) derived from fibroblasts from Werner syndrome patients, human embryonic stem cell (hESC) therapy (84–89).

The medical literature reports cases of scleroderma concomitant with phenylketonuria, an autosomal recessive metabolic disease caused by congenital phenylalanine hydroxylase deficiency (51). Korneich presented the case of an 18-month-old girl with sclerodermal lesions on the chest and proximal extremities of the lower limbs that improved after restriction to phenylalanine (37). The lesions are limited to the skin and subcutaneous tissue, respects the hands and forefoot and does not affect the internal organs (51). Hyperphenylalaninemia affects melanin synthesis and causes skin hypopigmentation (91). Histologically, the aggregation of l-phenylalanine is observed in the form of deposits of fibrils similar to amyloid (91, 154). The histological aspect shows the important increase of phenylalanine in the skin at the intracellular level and non-specific dermatitis (90). Pegvaliase injectable is an effective therapeutic option that lowers the concentration of l-phenylalanine, while the BH4 synthetic analogue sapropterin dihydrochloride registered good results only in some cases. A low-protein diet supplemented with amino acids without l-phenylalanine are recommended (91).

Cases of scleredema with proximal induration of the skin have also been reported in the context of β-hemolytic streptococcal infection, in which distal induration of the hands and feet is absent (37). Aichelburg and colleagues presented a case of scleredema adultorum Buschke installed 7 weeks after a streptococcal infection of the upper respiratory tract. The skin induration was located on the upper chest with bilateral symmetrical extension to the neck, face, shoulders and arms. The symptoms went away after antibiotic therapy and intravenous treatment with immunoglobulins (92). Radiation, vibration, trauma can cause scleroderma-like syndromes (89). Hashimoto and Craig observed that vibrations induce acrosclerosis. Histological examination shows excessive collagen deposition in the dermis, blood vessels walls and perivascular, numerous lysosomes in endothelial cells and degenerative changes in peripheral nerves, demyelination of axons, collagen deposition in endomysium and perimysium (93). Although not very clear, silicone implants may be involved in sclerodermal lesions (37). Sometimes the onset of hypothyroidism may be marked by the appearance of atypical skin lesions (37, 89). Although they look like very severe hyperkeratosis with skin hardening and discoloration of the skin, these lesions do not resemble those of lichen sclerosus and morphea. For this reason it is recommended to evaluate thyroid function in all cases of lichen sclerosus and morphea. For the differential diagnosis between sclerodermal and SSc lesions, the serological profile and the histological examination are not always conclusive, which is why the distribution, extent and depth of the skin lesion are taken into account (89, 94). Histologically, there is an accumulation of mucin, hyperplasia of fibroblasts with excessive collagen deposition (94). The substitution treatment improved the clinical signs (95). Sclerodermoid lesions have also been identified in chronic graft-versus host disease, an immunological reaction that may occur after transplantation of allogeneic hematopoietic stem cells (37, 51). The lesions first appear on the trunk in the form of plaques and then generalize. Marked extension of the skin lesions on the chest limit the expansion of the chest and causes restrictive lung dysfunction. The skin biopsy showed the thickening of the reticular dermis and the dermal papillae with collagen fibers arranged in the form of bundles and the increased presence of melanophages in the dermis (96). The immunosuppressive treatment with Azathioprine in combination with cortisone preparations has proven partially effective (96). 400 mg per day of imatinib mesylate, tyrosine kinase inhibitor, improved the skin lesion (97). Infiltration of the skin with neoplastic cells in the case of multiple myeloma is followed by sclerodermatous lesions (51). The administration of a proteasome inhibitor (ixazomib, bortezomib, carfilzomib) in combination with glucocorticoids, chemotherapy, immunomodulators has proven effective in improving the clinical aspects of the disease (98).

Cutaneous scleroatrophy of the hands and forefoot accompanied by sclerodactyly has been observed in scleroatrophic Huriez syndrome. This syndrome, also called palmoplantar keratoderma, is a rare congenital autosomal dominantly transmitted dermatosis. Studies have shown that scleroatrophic skin lesions are at risk of developing squamous cell carcinoma (or even basal cell carcinoma which has been found to occur on scar sites) (51, 99, 100). The histological examination of the skin lesion shows the almost complete absence of Langerhans cells in the epidermis, an aspect that suggests a neoplastic risk (101). In addition, discrete papillomatosis, acanthosis with irregular aspects, parakeratosis, hyperkeratosis, hypergranulosis are observed (102, 103). The treatment includes oral and topical retinoids, keratolytics and emollients with 20% urea applied locally (103).

## 3 Conclusion

This paper brings together the types of sclerodermal lesions known in the medical literature and practice and records the clinical and paraclinical features that distinguish them from localized and systemic scleroderma. The etiology remains unknown, although possible triggers and predisposing factors have been foreshadowed. Proper diagnosis is essential for proper management, given that some sclerotic lesions may show some improvement by controlling the underlying disease. However, the unknown multiples of the etiopathogenesis and the lack of a truly effective treatment necessitate further prospective studies for a better understanding of sclerodermal lesions.

## Author contributions

All authors made a significant contribution to the work reported, whether that is in the conception, study design, execution, acquisition of data, analysis and interpretation, or in all these areas; took part in drafting, revising or critically reviewing the article; have agreed on the journal to which the article has been submitted; and agree to be accountable for all aspects of the work. All authors contributed to the article and approved the submitted version.
